# Assessment of corrosion restraint effect of carbon steel immersed in hydrochloric acid by expired tilmicosin drug

**DOI:** 10.1038/s41598-025-21636-9

**Published:** 2025-10-28

**Authors:** Mahmoud A. Ali, Ahmed A. El-Hossiany, Abdelfattah M. Ouf, Abd El-Aziz S. Fouda

**Affiliations:** 1 Department of Chemistry, Faculty of Science, Mansoura University, Mansoura, 35516 Egypt; 2 Delta for Fertilizers and Chemical Industries, Talkha, 1179 Egypt

**Keywords:** Carbon steel, Corrosion inhibition, Expired tilmicosin drug, Hydrochloric acid, Langmuir isotherm, Theory and computation, Electrochemistry

## Abstract

This research investigates the application of Expired Tilmicosin Drug as a corrosion inhibitor for C-steel in a 1 M HCl solution. FT-IR measurements, atomic force microscopy (AFM), electrochemical impedance spectroscopy (EIS), weight loss (WL), and potentiodynamic polarization (PDP) were employed to evaluate the efficacy of Expired Tilmicosin Drug in protecting C-steel against corrosion. According to the findings, the inhibition efficiency (% IE) increased as the Expired Tilmicosin Drug concentration increased and reached 91.8% at 300 ppm, 25 °C, but decreased to 85.6% at 45 °C. The investigated drug acted as a mixed–kind inhibitor from the data of PDP technique. The Langmuir adsorption model was supported by the drug’s adsorption behavior on the C-steel surface. The adsorption phenomena was found to be spontaneous based on the computed values of the standard free energy change of adsorption (ΔG^o^_ads_). Fourier transform infrared spectroscopy (FT-IR) and atomic force microscopy (AFM) demonstrated that the drug molecules had a strong bond with the C-steel surface. Density functional theory (DFT) calculations and molecular dynamics (MC) simulations provided further insight into the chemical interactions between Expired Tilmicosin Drug and the C-steel surface. This study confirms that there is agreement between experimental and theoretical results. This research introduces the novel application of Expired Tilmicosin Drug, highlighting its non-toxic nature and cost-effectiveness, making it a promising alternative for corrosion prevention in industrial applications. This study investigates the dual role of Expired Tilmicosin Drug in addressing expired pharmaceutical waste and developing an efficient corrosion inhibitor for C-steel in acidic environments. Repurposing Expired Tilmicosin Drug provides a sustainable solution to environmental hazards while demonstrating high corrosion inhibition efficiency.

## Introduction

Carbon steel is a highly multifunctional material. Its numerous advantages include low cost, excellent mechanical properties, durability, strength, and versatility. These attributes make it ideal for diverse applications such as construction, chemical processing, fossil fuel industries, metal processing equipment, marine uses, nuclear power plants, mining, pipelines, and petroleum refining^1^. Evaluating C-steel corrosion, especially in acidic media, is crucial because acids are commonly used for pickling, cleaning, descaling, and oil well acidization^2^. Among acidic media, hydrochloric acid solution is more economical, efficient, and trouble-free than other mineral acids^3^. However, iron and its alloys can corrode during these applications, leading to resource waste^4^. Using inhibitors is one of the most popular methods of preventing corrosion, especially in acidic settings^5^. The most well-known acid inhibitors are organic molecules that contain nitrogen (N-heterocyclic), sulfur, long carbon chains, aromatic, and oxygen atoms. Among these, organic inhibitors have several advantages, such as high inhibitory efficiency, cheap cost, low toxicity, and ease of synthesis^6^. In various aqueous media, organic heterocyclic compounds have been employed to limit the corrosion of C-steel^7–12^, copper^13^, aluminium^14^, and other metals^15^. Protecting the metal surface was made simpler by drug adsorption^16^. It has been discovered that these drugs are quite effective at preventing metal corrosion. Despite extensive study on the use of drugs to prevent metal corrosion, nothing is known about the use of expired medications in this context. Certain medications, such as tetracycline, cloxcillin, azithromycin, ampiclox, ampicillin, and orphenadrine, have been shown to dramatically lower metal surface corrosion. The majority of authors concur that most medications can be made from natural substances and that they are inhibitors that can effectively compete with green corrosion inhibition. When selecting a corrosion-inhibiting compound, the following criteria are considered: (1) ease of synthesis and purification; (2) environmental benignity and necessity for organic reactions; (3) the presence of oxygen, sulfur, and nitrogen as active sites in drug molecules^17^; and (4) studies indicating that 90% of drugs remain stable long after their expiration dates^18^. In recent years, the expired drugs have been used as corrosion inhibitors for a range of metals due to its non-toxic properties and in most cases active substance of the expired drugs degrades only infinitesimally^19^. It is crucial to investigate the connections between corrosion inhibition and adsorption. High C-steel inhibition efficiency has been demonstrated by heterocyclic compounds in both HCl^20^ and H_2_SO_4_ solutions^21^. Its inhibition is facilitated by the drug molecules’ adsorption on the metal surface^22^. As an example, “Januvia gave 79.5% IE for Zn in HCl^23^, 2-mercaptobenzimidazole gave 82% IE for C-steel in HCl^24^, the antidiabetic drug Janumet gave 88.7% IE for mild steel in HCl^25^, chloroquine diphosphate gave 80% IE for mild steel in HCl^26^, Expired diclofenac sodium drug (DSD) yielded inhibition efficiencies of 99.99% and 83.32% in acidic and saline solutions, respectively, at a concentration of 150 ppm of DSD for aluminum alloy (Al6061) in 0.1 M HCl and 3.5% NaCl solutions^27^. In a 1 M HCl solution, tiazofurin was used as a new corrosion inhibitor for mild steel, with a maximum inhibition efficacy of almost 97% at 400 ppm^28^. In 15% HCl, the corrosion-inhibiting properties of expired metformin were evaluated for API X60 carbon steel. When KI was added, an inhibitory effectiveness of over 92% was observed at 60°C^29^. In a 1.0 M HCl solution, an expired herbal medication was used to prevent carbon steel from corroding. With increasing concentration, the inhibition efficiency increased, reaching 97.5% at 1.2 g/L of inhibitor^30^. There are financial and environmental benefits to using certain expired medications as potent inhibitors of metal corrosion. Ticarcillin and Carbenicillin, two selected expired medications, were therefore tested for their capacity to prevent aluminum corrosion in a 1.0 M HCl solution. At 298 K, the inhibitory efficiency (% IE) of Ticarcillin was found to be marginally greater (91%) than that of Carbenicillin (89%)^31^. Curam medication was used to protect stainless steel 304 in a 2.0 M HCl solution by acting as an environmentally friendly corrosion inhibitor. At a temperature of 298 K and a concentration of 300 ppm, Curam’s inhibitory efficiency was maintained at about 82%^32^. It was examined how the medication Dexamethasone (DM) affected mild steel corrosion in a 2 M HCl solution^33^. Using Weight Loss (WL), Electrochemical Impedance Spectroscopy (EIS), and Potentiodynamic Polarization (PDP) methods, the inhibitory efficiency (IE%) of DM was shown to be 80%, 81%, and 83% at a concentration of 0.4 g/L, respectively. Singh et al.^34^ explored the application of expired atorvastatin (EA) for inhibiting the corrosion of mild steel in a 1 M HCl solution, reporting an inhibition efficiency of 99.08% at a concentration of 150 ppm and a temperature of 25°C. An approach for using unused or expired paracetamol and carbamazepine pills as corrosion inhibitors for metals in various media is presented in this work^35^. In a 0.1 **M** sulfuric acid solution, carbamazepine showed a 90% corrosion inhibition efficiency on carbon steel, whereas paracetamol showed a 95% corrosion inhibition efficiency in acetic acid (0.25 **M**)”. A prospective corrosion inhibitor for mild steel in 1.0 M HCl was studied using an unused expired amiodarone (EAD) drug^36^. It showed outstanding inhibition at a concentration of 0.001 M, with an inhibition efficiency of 88.77%. In one study, Abdel-Hameed et al.^37^ utilized expired water-soluble lactulose as a corrosion inhibitor for a carbon steel electrode in a 1.0 M HCl acidic environment. Their weight loss method revealed a peak inhibitory efficiency of 97% with 300 ppm of the expired lactulose medication. In a distinct investigation, the corrosion inhibition efficacies of two porphyrin molecules, 5,10,15,20-tetra-[m-(methoxy)phenyl] porphyrin (m-TMPP) and 5,10,15,20-tetra-[p-(methoxy)phenyl] porphyrin (p-TMPP), were evaluated on copper alloy (C12510) in 1.0 M H_2_SO_4_ solutions^38^. Using the potentiodynamic polarization technique, the inhibition efficiency reached 96.23% for m-TMPP and 97.61% for p-TMPP. Separately, Al-Gorair et al.^39^ evaluated Salbutamol, effectively extracted from expired Farcolin, as an inhibitor against carbon steel (CSt) dissolution in 1.0 M HCl solution. Its inhibition efficiency reached 89.65% at a 200 ppm dose, determined by EIS technology.

Since most active pharmaceutical substances are significantly more expensive than current organic inhibitors, our study focused on repurposing expired or unused drugs (often from patient non-compliance). These drugs contain active inhibitory compounds, offering a dual benefit: reducing environmental contamination from pharmaceutical waste and lowering disposal costs for expired medications. Expired Tilmicosin Drug is water insoluble. The chemical nature of Expired Tilmicosin Drug is that of a macrolide structure derived from tylosin, with its cost not being readily available but subject to production methods. It is stable under normal conditions but sensitive to light and reactive with strong oxidizing agents. Its solubility is highly pH-dependent, being low in water but highly soluble in many organic solvents. A key aspect of its uniqueness is its ability to concentrate in and be retained by swine phagocytes, enhancing its effectiveness against bacterial infections. Research reviewed by Tanwer et al.^40^ regarding the use of drugs as corrosion inhibitors for metal surfaces found inhibition efficiencies ranging from 44.33% to 100% across various drugs, metals (steel alloys, aluminum, copper, zinc), and acids (HCl, H_3_PO_4_, etc.). A substantial number of these values were around 80% or 90%, with some also appearing near 70%. Consequently, Expired Tilmicosin Drug’s performance aligns with a significant protection level (90.2% – 92.2%) when evaluated against existing literature.

Tilmicosin may be studied experimentally for corrosion inhibition due to its chemical structure. These are typically niche, academic uses aimed at exploring environmentally friendly inhibitors. Some researchers explore this compound as eco-friendly alternatives to traditional synthetic corrosion inhibitors, due to: Multiple oxygen and nitrogen atoms, a large molecular size that could adsorb well onto metal surfaces and surfactant-like properties.

The current study focused on using the WL approach, PDP analysis, and EIS measurements to evaluate the IE of Expired Tilmicosin Drug on C-steel. This study also focused on using atomic force microscopy (AFM) and scanning electron microscopy–energy dispersive spectroscopy (SEM–EDAX) to assess the surface morphology of carbon-steel. The experimental methodology part goes into depth on the preparation of the specimen and solution as well as the instruments, and the results and discussion section presents the findings that were observed.

## Experimental techniques

### Preparation of specimen

Carbon-steel specimens measuring 2 × 2 × 0.2 cm² and containing 0.2% C, 0.003% Si, 0.35% Mn, 0.025% Si, 0.02% P, and the rest Fe by weight% were polished to a mirror-like surface, then degreased with acetone before being used in the weight loss (WL) investigation. Additionally, surface analysis, electrochemical impedance spectroscopy, and potentiodynamic polarization were performed on a specimen area of 1 cm².

### Preparation of solutions

The acid solution (1.0 M HCl, 37%) was procured from El-Nasr Company, “Egypt and formulated through the dilution of analytical grade HCl utilizing double distilled water. All experiments were performed at varying temperatures and without any mechanical agitation. The expired Tilmicosin Drug inhibitor stock solution was prepared by dissolving one gram of the pharmaceutical in a minimal amount (10 mL) of dimethyl formamide (DMF), then completing the volume to one liter with ethanol to yield a 1000 ppm stock solution. This stock was subsequently diluted with doubly distilled water to achieve the desired concentrations (50–300 ppm). Expired Tilmicosin drug is an organic composite, which have the chemical formula C_24_H_20_N_6_O_3_ and purchased from Sandozinc and Pfizer inc companies was utilized here as inhibitor. Expired Tilmicosin Drug is a macrolide antibiotic. Micotil is the brand name under which it is supplied. It is used in veterinary medicine for the treatment of bovine respiratory disease and enzootic pneumonia caused by Mannheimia (Pasteurella) haemolytica in sheep”^41^.

**Figure:**
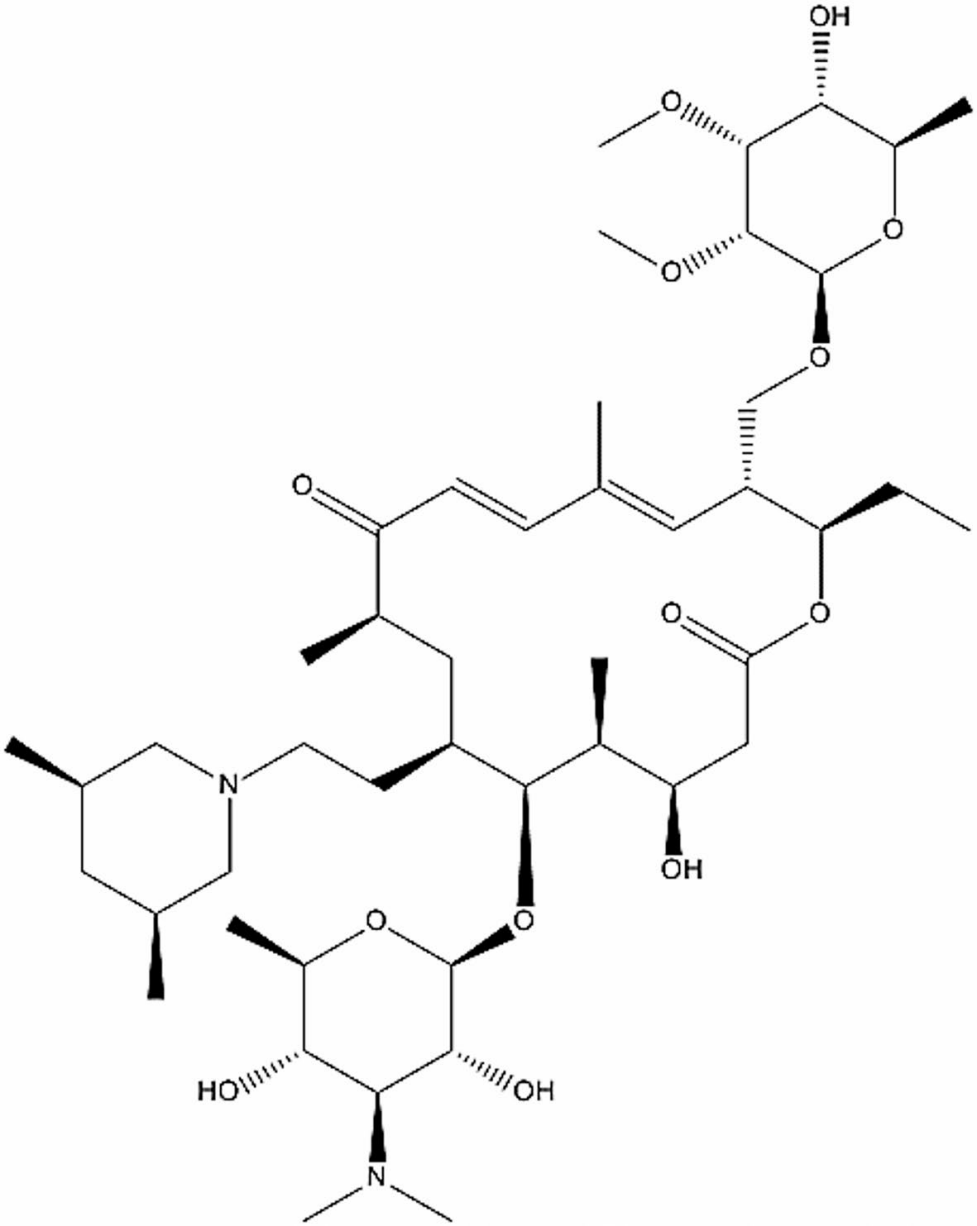
Tilmicosin drug, chemical formula: C_46_H_80_N_2_O_13_, molecular weight: 868.57.

### Determination of corrosion rate (CR) via WL method

C-steel specimens for the WL investigation were made in compliance with ASTM G31-72 guidelines^42^. The specimens were suspended using glass hooks and subjected to exact quantification in order to estimate the corrosion rate (CR). Both with and without the Expired Tilmicosin Drug, the specimens’ initial weight was recorded before they were submerged in a 100 mL test solution in a beaker. Expired Tilmicosin Drug, inhibitor doses ranging from 50 ppm to 300 ppm were assessed after a preliminary weight loss (WL) experiment was conducted. For three hours, the samples were kept immersed in each test solution. After the three-hour immersion, the samples underwent a series of stages that included cleaning, drying, and then reweighing; each step was carried out in triplicate. The following mathematical formulae were used to calculate the corrosion rate (k_corr_), which was measured in W, and to quantify and express the inhibition efficiency (IE) as a percentage^43^:

1 $$\%\:{\it IE=\theta\:{x}\: 100=[1- (\Delta\:W/\Delta W^{\circ})] \times 100}$$

Where, ΔWº and ΔW are the WL in attendance and non-attendance of various doses of the Expired Tilmicosin Drug,. The corrosion rate equation is given by:

2 $${\rm k_{corr} = (\Delta\:W / A) / (\rho\:t)}$$

where A is the specimen’s surface area (cm^2^), ρ is the metal’s density (g/cm^2^), t is the exposure period (min), and ΔW is the WL (mg). The measurements were conducted without stirring or flow.

### Electrochemical measurements

In electrochemical measurements, two methods were used Tafel polarization (PDP) and impedance (EIS). The Gamry (PCI4/750) Potentiostat/Galvanostat/ZRA. https://www.gamry.com/ support-2/technical- support/installation- and- setup/insta llingechem-analy st) Before beginning any method, the working electrode was polished using emery papers^44^. Three-electrode electrochemical cells were used for the polarization analysis. A low-density metallic substrate was used to create the specimen, which had a cathode surface area of 1 cm² on one face and a red enamel layer on the other face. The reference electrode was a saturated calomel electrode (SCE), and the counter electrode was a rectangular piece of platinum foil. A substantial cathode formed as a result of the counter electrodes experiencing a big environmental change. To get rid of any possible inhibitory chemicals, the working electrode and platinum counter electrode were submerged in distilled water. A saturated calomel electrode (SCE) was used as the reference electrode in the experiment. Tafel polarization tests were performed at a scan rate of 0.2 mV/s within the potential range of ± 250 mV with respect to the open circuit potential (OCP). To reduce experimental errors, each test was conducted three times, and the temperature was continuously kept at 25 °C throughout the trials. The following formula can be used to calculate the inhibitory efficiency (IE).:

3 $$\% \:IE_{PDP} = 100 \:x\: [1- (i_{corr.} /i^{o}_{corr.})]$$

The symbols i_corr_ and i^o^_corr_ represent the corrosion current densities with and without Expired Tilmicosin Drug, respectively.

EIS tests were performed in the frequency range from 100 kHz to 0.01 Hz, with an excitation signal provided by a 10 mV sine wave voltage. The calculation of IE detected by using Eq. 4:

4 $$\%\:IE_{EIS}=R_{ct}-R_{ct0}/R_{ct}\:x\:100$$

“Where 𝑅_ct_ and 𝑅_ct0_ are charge transfer resistances with and without Expired Tilmicosin Drug. The measurements were carried out without stirring or flow as in the weight loss.

### Micromorphology analysis (AFM, FTIR)

Using C-steel samples, the corrosion’s morphology was also investigated. Both the C-steel samples and those without the addition of 300 ppm of Expired Tilmicosin Drug, were submerged in HCl. After soaking for a certain amount of time, the samples were taken out and repeatedly cleaned with ethanol and double-distilled water. Following drying, the corrosion morphology was investigated using Pico SPM2100 AFM equipment for AFM testing, which were used to assess the film’s surface roughness both with and without Expired Tilmicosin Drug, (Nanosurf C3000 Software Version 3105) https://www.nanosurf.com/en/software/c3000-control-software. FT-IR spectra were obtained by identifying peaks linked to particular functional groups in the inhibitor’s chemical composition using Attenuated Total Reflectance Fourier Transform Infrared Spectroscopy (ATR-FTIR), a device produced by Thermo Fisher Scientific in Waltham, Massachusetts, USA. These surface characteristics were determined in order to support the electrochemical findings.

## Theoretical calculations

### Quantum chemical calculation

Researchers used Materials Studio version 7.0, a software suite, for the chemical analysis. This software simulates a variety of materials using a semi-empirical method based on Density Functional Theory (DFT). “Materials Studio is an effective tool that includes features for computational chemistry, molecular dynamics, bioinformatics, chemical informatics, and quantum mechanics.(Material Studio software (version 7.0) https://www.fullversiondl.com/accelrys-materials-studio-v7-0/) Version 7.0 was used in this work to carry out sophisticated research on the materials of interest, such as carbons and polymers. Trajectories were calculated in a semiempirical manner. In the default configuration, molecules were categorized using the DNP function and the B3LYB function (Becke-3-Parameter-Lee-Yang-Parr). In order to influence purification through COSMO, water creation and the efficient usage and decrease of Earth’s orbits were employed as solutions.

### Monte‑Carlo simulations (MC)

Monte Carlo simulations were used to examine the interaction between the investigated chemicals and the surface of the Fe (1 1 0) plane. It is crucial to investigate the adsorption of neutral and protonated inhibitor molecules in the presence of water molecules, which compete for active sites on the surface of iron (1 1 0), as the inhibitors under research have decreased the corrosion of carbon steel in acidic solutions. One hundred water molecules were added as co-adsorbates in order to achieve this goal. First, the citation module was used to optimize the geometry of each of the inhibitor and water molecules.

## Results and discussion

### WL tests

**Fig. 1:**
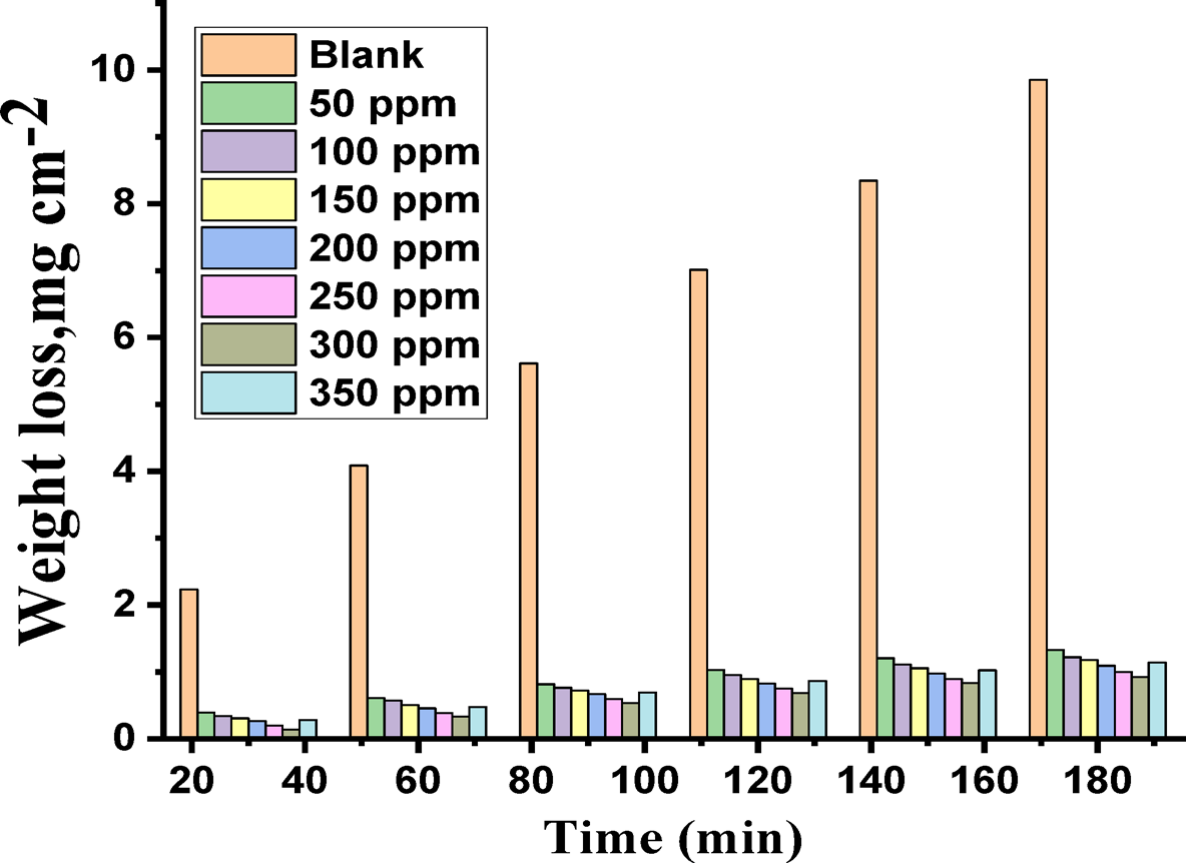
Time vs. WL diagrams of C-steel in 1 M HCl without and with altered doses of expired tilmicosin drug at 25^o^ C.

The weight loss (WL) curves for C-steel submerged in a 1 M HCl solution with and without Expired Tilmicosin Drug, are shown in Fig. 1. “We used these curves to determine the activation energy (˛E^*^), inhibition efficiency (% IE), and corrosion rate (k_corr_) for C-steel at different temperatures between 25 °C and 45 °C^45^. More than 45 °C the results were not good and unstable because high temperatures accelerate the chemical degradation of the drug, leading to a loss of potency and potentially increasing its toxicity. For Tilmicosin, a specific study showed significant drops in its activity after exposure to 45 °C for 24 hours, but the thermal stability of an expired drug isn’t guaranteed. A summary of the information gathered is given in Table 1. The table demonstrates a clear trend: the corrosion rate falls with increasing temperature, while the inhibition efficiency grows as Expired Tilmicosin Drug, dosage increases. The incorporation of the heteroatom onto the metallic surface of the C-steel was credited with the increased corrosion inhibition efficacy. The results showed that Expired Tilmicosin Drug, has advantageous anti-corrosive properties. The findings of this study were consistent with those of previous studies involving different medicinal goods that had expired^46–49^. This action is brought on by the drug’s active molecules adhering to the C-steel surface. The maximum IE of 93.2% was attained at a dose of 300 mg/L. k_corr_ increases as the immersion period is lengthen at a fixed dose of the drug and with rise in temperature”.

**Table 1:** Outcome data of WL measurements of C-steel at temperatures (25–45 °C) at 120 min dipping in the presence and absence of altered doses of expired Tilmicosin Drug.

Temp., C	[inh], ppm	CR, mg cm^−2^ min^−1^	Ө	% IE
25	Blank	0.069	-----	-----
	50	0.0088	0.873	87.3
	100	0.0077	0.888	88.8
	150	0.0072	0.895	89.5
	200	0.0071	0.897	89.7
	250	0.0065	0.906	90.6
	300	0.0057	0.918	91.8
	350	0.0072	0.896	89.6
30	Blank	0.087	-----	-----
	50	0.0124	0.858	85.8
	100	0.0114	0.869	86.9
	150	0.0110	0.873	87.3
	200	0.0098	0.887	88.7
	250	0.0089	0.898	89.8
	300	0.0085	0.902	90.2
35	Blank	0.1100	-----	-----
	50	0.0204	0.815	81.5
	100	0.0190	0.827	82.7
	150	0.0182	0.835	83.5
	200	0.0155	0.859	85.9
	250	0.0152	0.862	86.2
	300	0.0131	0.881	88.1
40	Blank	0.1550	-----	-----
	50	0.0309	0.801	80.1
	100	0.0290	0.813	81.3
	150	0.0267	0.828	82.8
	200	0.0250	0.839	83.9
	250	0.0239	0.846	84.6
	300	0.0209	0.865	86.5
45	Blank	0.1770	-----	-----
	50	0.0370	0.791	79.1
	100	0.0338	0.809	80.9
	150	0.0329	0.814	81.4
	200	0.0308	0.826	82.6
	250	0.0285	0.839	83.9
	300	0.0255	0.856	85.6

### Adsorption isotherms

**Fig. 2:**
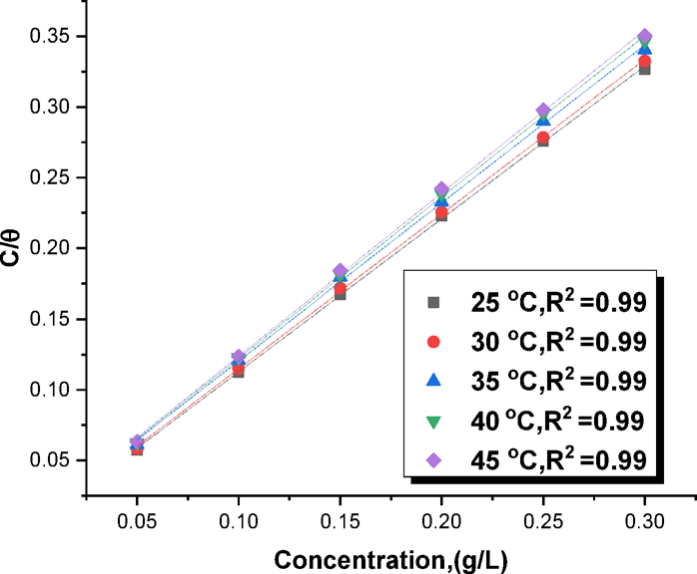
curves of langmuir adsorption of c-steel without and with altered doses of expired tilmicosin drug.

For the drug to exert its inhibitory effect on the corrosion of C-steel, “there must be an adsorption of the Expired Tilmicosin Drug, molecules onto the metal surface, resulting in increased surface coverage as the initial stage of the inhibition mechanism. The values representing the degree of surface coverage (Θ), defined as the proportion of the C-steel surface occupied by the inhibitor, are considerably valuable for ascertaining the inhibitor’s adsorption characteristics. Assuming a direct correlation between surface coverage and inhibition efficiency, the surface coverage can be determined from the expression: IE% = Θ x 100. The experimental data underwent an fitting process with several established isotherm models, including Langmuir, Temkin, Frumkin, and Freundlich. The correlation coefficient (R²) was the metric used to ascertain which of these isotherms most accurately represented the observed data. It was determined that the Langmuir isotherm provided the optimal fit for the tested material’s collected data, evidenced by an R² value exceeding 0.99 (as depicted in Fig. 2). The standard mathematical representation of the Langmuir isotherm is presented in reference^50^.

5 $$C/ \ominus= 1/K_{ads}+C$$

A Langmuir adsorption isotherm was constructed using the weight loss data, enabling the computation of thermodynamic parameters. Drug particles cover a monolayer on the C-steel surface^51,52^, and we depict the linear relationship between C/ϴ and C with slope close to unit (Fig. 2) using the Langmuir isotherm explained by Eq. 5. The adsorption parameters, ΔG^0^_ads_ and K_ads_, were determined using Eq. 6.”^53^.

6 $$\Delta\:G^{o}_{ads}=RT\: ln(K_{ads} x 55.5)$$

Table 2 presents the values of ΔG°_ads_ (standard Gibbs free energy of adsorption) as a function of temperature, averaged over the range of 298–318 K. The ΔH_ads_ (standard enthalpy of.

adsorption) value was determined using the Van’t Hoff equation:

7 $$\:{{log\:K}}_{{ads}}{=}\frac{{-}{\Delta\:H}{ads}}{{2.303RT}}{+constant}$$

**Fig. 3:**
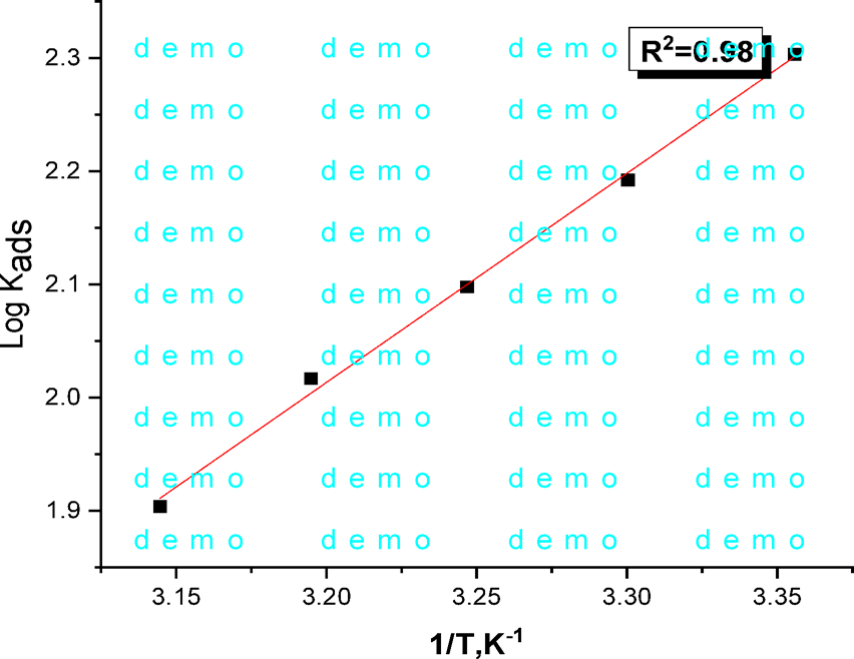
Plot of log K_ads_ vs. 1/T for the adsorption of expired tilmicosin drug on C-steel in 1 M HCl.

“A linear relationship was observed when plotting log K_ads_ against 1/T (Fig. 3). The slope of this line allowed for the determination of ΔH_ads_. By combining the calculated values of ΔG°_ads_ and ΔH°_ads_, the entropy of adsorption (ΔS_ads_) was determined at all investigated temperature”.

8 $$\:{\Delta\:S}{ads}{=\:}\frac{\varDelta\:Hads{-\:}{{\Delta\:G}}_{{ads}}^{{o}}}{{T}}$$

**Table 2:** Langmuir parameters of expired tilmicosin drug on C-steel surface by Langmuir isotherm.

Temp.,ᵒC	K _ads,_ L/mg	-ΔGᵒ_ads,_ kJ mol^−1^	-ΔH _ads,_ kJ mol^−1^	ΔS _ads,_ J mol^−1^K^−1^
25	201.2	23.1	35.0	77.4
30	155.8	22.8		75.3
35	125.3	22.7		73.5
40	103.9	22.6		71.9
45	80.1	22.2		69.7

The adsorption parameters ∆G°_ads_ and K_ads_ (Table 2) were found to decrease with temperature. The negative value of ∆G°_ads_ indicates that the adsorption process is spontaneous and the stability of the adsorbed layer on C-steel. The ∆G°_ads_ values, which range from 22.2 to 23.1 kJ mol^−1^, suggest that the drug is chemically and physically adsorbed onto the C-steel surface^54^. The negative sign of ∆H_ads_, which indicates an exothermic adsorption process, suggests that the drug was adsorbed onto the C-steel surface either physically or chemically”^55,56^. The adsorption of Expired Tilmicosin Drug, molecules onto the C-steel surface is primarily a physical adsorption.

process, evident from the ΔHads​ value being less than 41.8 kJ mol⁻¹. This suggests weak interactions, mainly electrostatic forces between opposing charges on the Expired Tilmicosin Drug, molecules and the high-energy C-steel surface. The lone pairs of electrons on nitrogen and oxygen atoms, alongside the increased electron density of the benzene ring within the Expired Tilmicosin Drug molecule, enhance its adsorption onto the C-steel surface. This occurs predominantly through electrostatic attraction, rather than electron transfer, a conclusion supported by the negative ΔG^o^_ads_​ value.

### Activation parameters

To clarify the mechanism of the response between electrostatic discharge (Expired Tilmicosin Drug) and the C-steel surface, activation parameters were used. This process was interpreted using the Arrhenius and transition state equations (Eqs. 9 and 10).

9 $${\rm k_{corr}=A e^{- E*a/RT}}$$

10 $${\rm k_{corr}=RT/Nh \:e^{(\Delta\:S^{*}/R)}e^{(-\Delta\:H^{*}/RT)}}$$

**Fig. 4:**
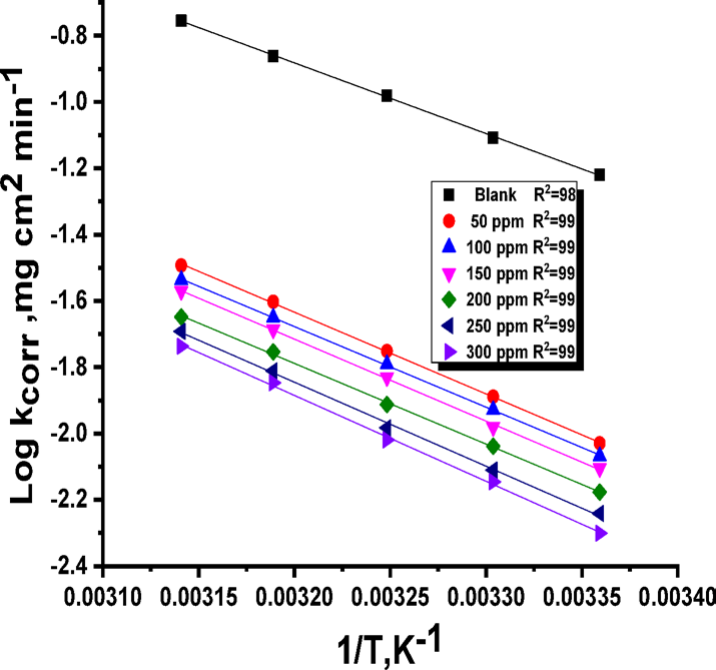
log k_corr_ vs. 1/T for C-steel in with and without expired tilmicosin drug in1M HCl.

**Fig. 5:**
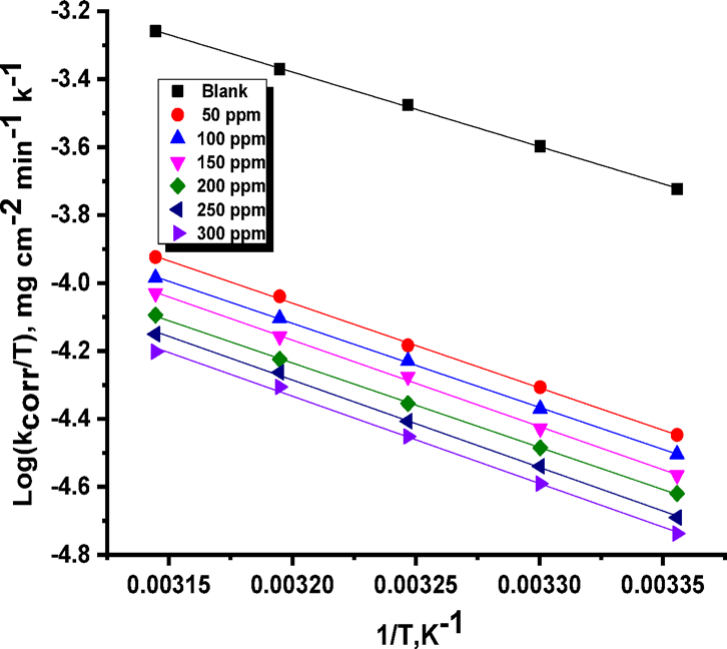
log k_corr_/T vs. 1/T for C-steel without and with expired tilmicosin drug in 1 M HCl.

E^*^_a_, ΔH^*^, and ΔS^*^ stand for activation energy, enthalpy, and entropy, respectively. “These kinetic parameters are shown by the straight line that results from plotting log k_corr_ against 1/T (Fig. 4)^57^. The slope of this line indicates the activation energy (E^*^_a_). The values of ΔH^*^ and ΔS^*^ are obtained by a linear relationship between log k_corr_/T and 1/T (Fig. 5). The presence of the Expired Tilmicosin Drug (likely an inhibitor) leads to an increase in the observed activation energy (E_a_^∗​^) for corrosion (Table 3). This suggests that Expired Tilmicosin Drug molecules create an adsorbed layer, acting as a barrier film on the C-steel surface. Consequently, the aggressive medium must overcome a higher energy hurdle before it can make contact with and corrode the metal. This finding supports a physical adsorption mechanism for the Expired Tilmicosin Drug. Furthermore, the positive values for the enthalpy of activation (ΔH^∗^) indicate that the corrosion process itself is endothermic, meaning it absorbs energy from its surroundings. The negative entropy of activation (ΔS^∗^) indicates that the association process is favored over dissociation in the rate-determining step. This implies a decrease in disorder during the transition from reactants to the activated complex. However, in the presence of an inhibitor, the entropy increases, suggesting an occurrence of disorder. This observed disorder may stem from the adsorption of inhibitor molecules onto the metal surface, a process that necessitates the displacement of previously adsorbed water molecules. This displacement likely involves an initial phase of disorder before the inhibitor’s adsorption leads to a more ordered arrangement on the surface.

**Table 3:** Activation parameters of C-steel in presence and absence of expired Tilmicosin drug in 1 M HCl.

Conc., ppm	E_a_^*^, kJ mol^−1^	∆ H^*^, kJ mol^−1^	-∆S^*^, J mol^−1^K^−1^
1 M HCl	39.9	37.3	137.1
50	54.6	52.2	77.6
100	56.8	54.5	73.2
150	57.9	55.3	71.2
200	58.3	55.9	65.2
250	60.5	58.2	60.5
300	61.9	59.5	58.6

### PDP tests

**Fig. 6:**
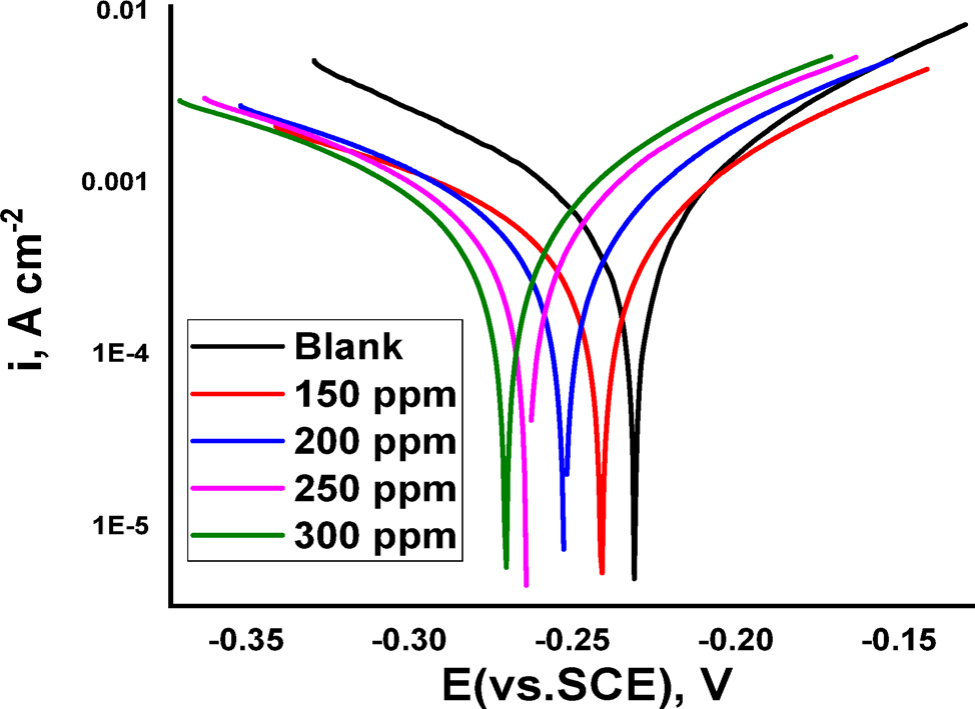
PDP of C-steel dissolution in the attendance and absence of expired tilmicosin drug in 1 M HCl.

PDP curves were produced for C-steel in an HCl atmosphere with and without different Expired Tilmicosin Drug doses. The cathodic curves in Fig. 6 shows the features of a Tafel line, indicating that a pure activation mechanism drives the H⁺ reduction reaction on the steel surface^58^. Both the anodic and cathodic current densities significantly decreased when Expired Tilmicosin Drug was added to the acidic solution, as seen in Fig. 6. Table 4 lists the Tafel Polarization (PDP) parameters, which include i_corr_, E_corr_, β_c_, β_a_, IE, and θ. According to Table 1’s results, the corrosion current density (i_corr_) is lower when Expired Tilmicosin Drug is present than when it is not. This suggests that Expired Tilmicosin Drug first adsorbs onto the C-steel surface before using its active sites to create a straightforward blocking mechanism that provides protection. Moreover, Expired Tilmicosin Drug has a greater effect on the cathodic and anodic reactions when β_c_ and β_a_ values show only minor changes. The observation that the Tafel slopes do not display a consistent behavioral pattern relative to the blank solution further supports the assertion that the adsorption process of Expired Tilmicosin Drug on the C-steel surface in a 1 M HCl solution is attributed to the blockage of active sites by the Expired Tilmicosin Drug molecules and indicate the mixed type of inhibitor. As the concentration of the Expired Tilmicosin Drug increases, both the inhibition efficiency (IE) and the surface coverage (θ) also rise. Crucially, the displacement of the corrosion potential (E_corr_​) remains below 85 mV across all tested concentrations. This observation, combined with the reduction in both cathodic and anodic partial currents, signifies that the Expired Tilmicosin Drug acts as a mixed-type inhibitor. However, the data further suggests that under the experimental conditions, the inhibitor primarily exerts a cathodic effect.

**Table 4:** PDP parameters of C-steel in dissimilar doses of expired Tilmicosin drug.

Conc., mol Lˉ¹	-E_corr_, mV (mVvs.SCE)	β_a_, mV decˉ¹	-β_c_, mV decˉ¹	i_corr_, µA mˉ²	θ	% IE_PDP_
Blank	223	99	156	1080	----	----
150	224	138	205	233	0.784	78.4
200	225	114	246	198	0.816	81.6
250	226	122	234	156	0.855	85.5
300	227	130	219	105	0.902	90.2

### EIS method

**Fig. 7:**
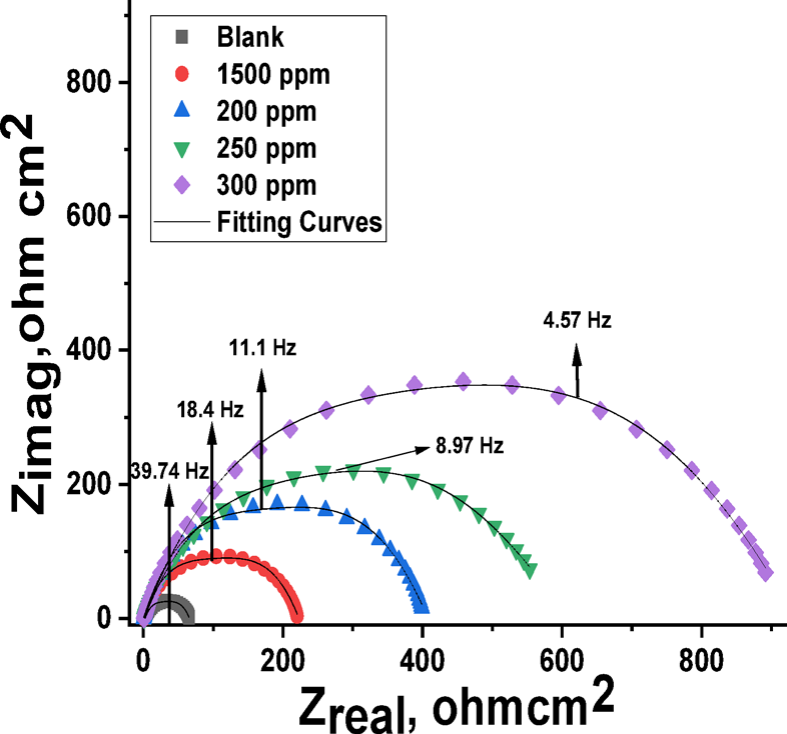
Nyquist diagram of C-steel with and without altered doses of Expired Tilmicosin Drug in 1 M HCl.

**Fig. 8:**
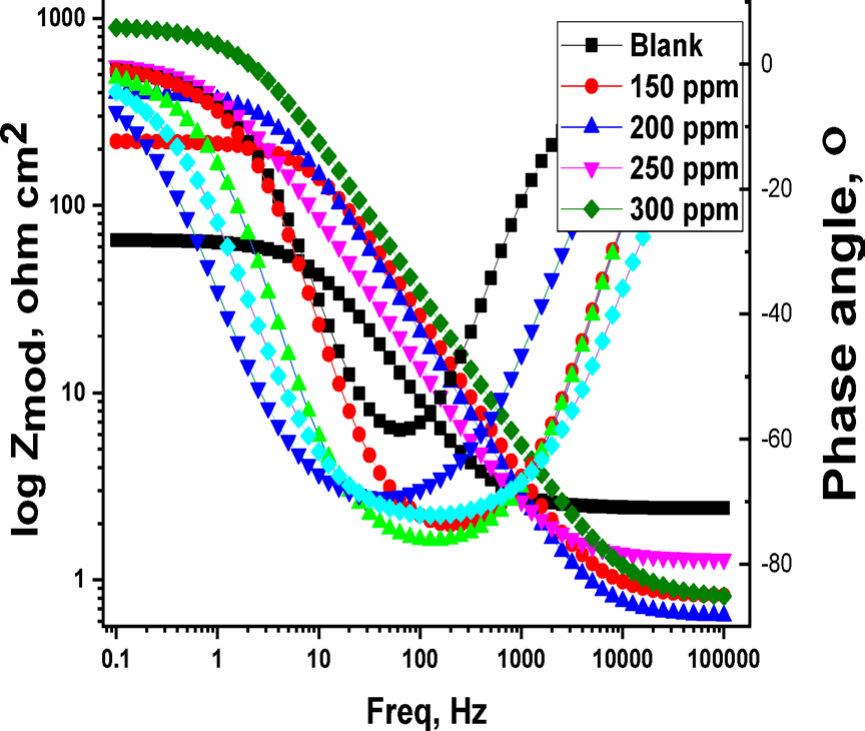
Bode diagram for C-steel dissolution with and without altered doses of.

**Fig. 9:**
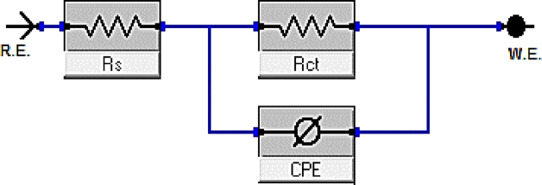
Simple circuit utilized to fit the EIS outcomes.

Figures 7 and 8 illustrate the Nyquist and Bode plots obtained for C-steel in a 1 M HCl solution containing varying concentrations of Expired Tilmicosin Drug at 25 °C. “The impedance spectra of the C-steel exhibit a low-frequency inductive loop and a high-frequency capacitive loop. The diameter of the capacitance loop shows a significant increase with increasing concentrations of the Expired Tilmicosin Drug drug within the corrosive medium (Fig. 7). This observation suggests that the antibiotic Expired Tilmicosin Drug, which demonstrates a substantial inhibitory effect on C-steel corrosion, undergoes adsorption and consequently elevates the charge transfer resistance (R_ct_). As the concentration of the drug in the solution is elevated, the diameter of the semicircle expands. This phenomenon indicates that the electron transfer step constitutes the rate-determining step throughout the corrosion process. Consistent with the predictions of EIS theory, the loops observed in the high-frequency region do not represent ideal semicircles. This deviation can be attributed to the non-ideal behavior of the electrical double layer as a perfect capacitor. The observed imperfect effectiveness is ascribed to frequency dispersion, which arises from the inherent roughness and non-homogeneity of the electrode surface. While Fig. 8 presents the Bode plots, the capacitive reactance modulus in these plots exhibits an increase by an order of magnitude within the low-frequency region, as depicted in Fig. 8. Furthermore, the slope of the impedance modulus approaches unity, and the phase angle reaches − 75◦ in the intermediate frequency range. This observation suggests the presence of a significant capacitive behavior associated with the Expired Tilmicosin Drug on the C-steel. The adsorption of Expired Tilmicosin Drug onto the C-steel surface results in the creation of a single time constant, as evidenced in Fig. 8 by the presence of only one peak in the phase angle plot. The semicircle Nyquist plot diagram’s diameter is increased by increasing the Expired Tilmicosin Drug dosage^59^. By fitting the result data to an equivalent circuit, the Nyquist bends were investigated (Fig. 9). EIS characteristic are listed in Tables 5 and include R_ct_ (charge transfer resistance), C_dl_ (capacitance double layer), ˛ (surface coverage area), n (is a measure that reflects a deviation from ideal behavior ranged from − 1 to 1) and IE (inhibition efficiency). By improving the Expired Tilmicosin Drug concentration, the data of R_ct_ and % IE raised, due to the increase in the thickness of the adsorbed layer but the value of the C_dl_ decreases due to the increase in the thickness of double layer and/or the lowering in local dielectric constant”^60^. The estimated value of Y_o_ for the blank solution is greater than that of the inhibited solution, suggesting that Expired Tilmicosin Drug molecules interacted with the electrode surface. The reduction in the values of (n) in the inhibited solution compared to its absence is attributed to the heterogeneity of the C-steel surface^61^. The chi-squared was utilized to appraise the precision of the fitting outcomes, the small chi-squared values (Table 5) acquired for all the outcomes show that the fitted results have a great concurrence with the experimental findings.

**Table 5:** EIS parameters of C-steel with and without various doses of expired Tilmicosin drug in 1 M HCl.

[Inh] ppm	*n*	Y_o_, (µΩ^−1^ s^*n*^ cm^−2^)	*R*_ct_ Ω cm^2^	C_dl_ µFcm^−2^	θ	%IE	Goodness of fit (χ2)
**Blank**	0.879	418	63	253	----	----	19.78 × 10^−3^
**150**	0.869	198	220	123	0.714	71.4	16.47 × 10^−3^
**200**	0.861	167	401	107	0.842	84.2	19.89 × 10^−3^
**250**	0.851	162	634	102	0.901	90.1	17.67 × 10^−3^
**300**	0.848	141	807	98	0.922	92.2	18.49 × 10^−3^

Expired Tilmicosin Drug.

### Surface examination

#### AFM study

**Fig. 10:**
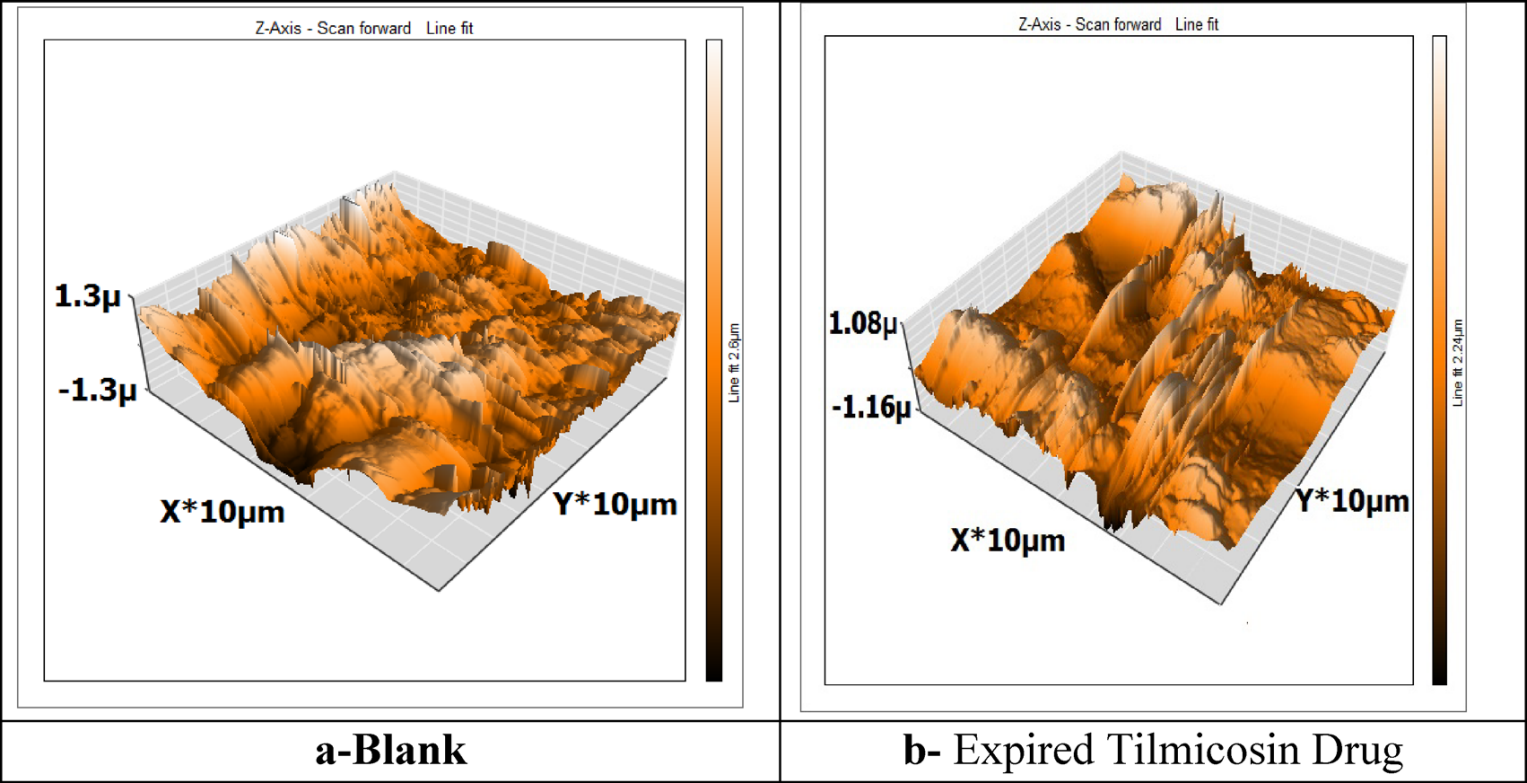
AFM (3D) of C-steel surface in existence and absence of expired tilmicosin drug in 1 M HCl. (Nanosurf C3000 Software Version 3105) https://www.nanosurf.com/en/software/c3000-control-software (These images were drawn by **Ahmed A. El-Hossiany his email is: dr.ahmedselm@gmail.com)**.

AFM is one of the best methods for figuring out how Expired Tilmicosin Drug impacts C-steel surfaces since it looks at the surface’s morphology. To ascertain whether or not 300 ppm Expired Tilmicosin Drug was present, the C-steel pieces were submerged in HCl solution for 24 h and the C-steel surface was polished before this examination started^62^. The topographic maps of the C-steel surface, which include 3D images, are displayed in Fig. 10. Table 6 contains AFM data, such as R_a_ (average roughness) and Rq (square roughness). R_a_ had a high value while C-steel was in HCl solution and a low value when it was free; however, it decreased when Expired Tilmicosin Drug was introduced.

**Table 6:** AFM data for C-steel surface.

specimen	C-steel in 1 M HCl	C-steel in presence of Expired Tilmicosin Drug
Rq (nm)	463	233
Ra (nm)	378	211

### ATR-FTIR analysis

**Fig. 11:**
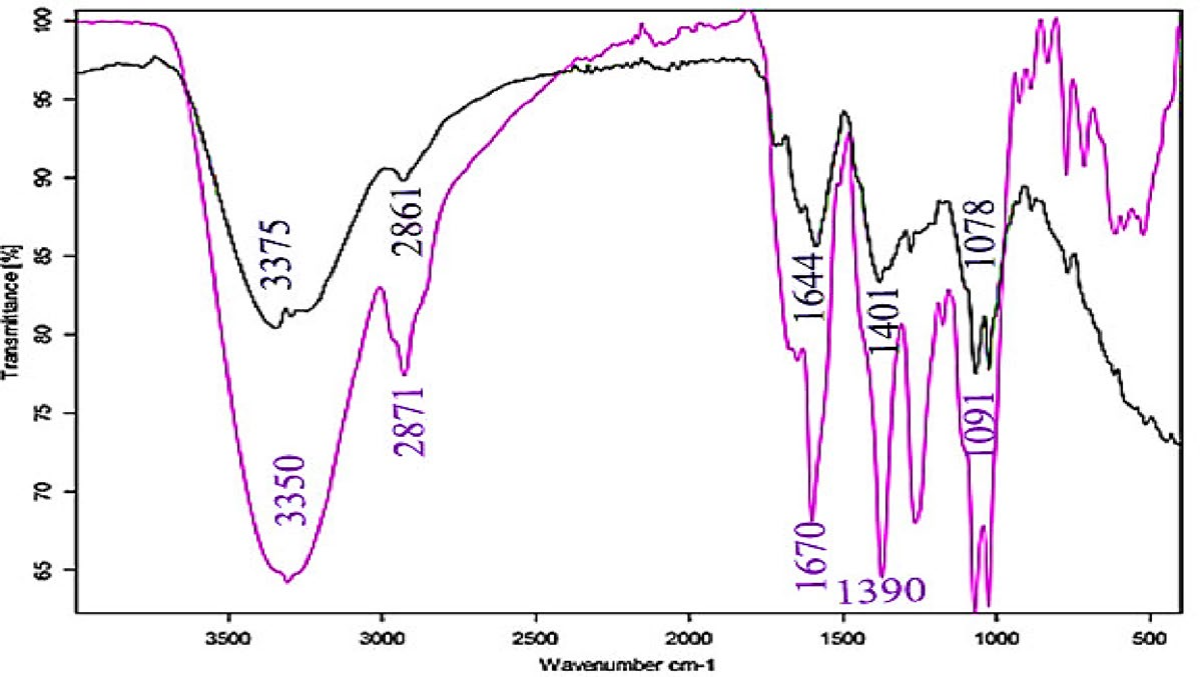
ATR- FTIR spectra of CS in existence and nonexistence of expired tilmicosin drug.

Potential interactions between the adsorbed inhibitor molecules and the carbon steel surface in the acidic environment were examined using FTIR analysis. “The functional groups found in the inhibitor molecules that are adsorbed onto the metal surface are usefully revealed by FTIR spectra. The FTIR spectra of pure Expired Tilmicosin Drug and Expired Tilmicosin Drug adsorbed on the surface of C-steel are shown in Fig. 11. Each peak on the spectrum has a defined value to facilitate the definition of the effective groups. Additionally, it is employed to determine the type of reaction that takes place between the Expired Tilmicosin Drug and the C-steel surface^63^. C-steel specimens were polished and submerged in a 1 M HCl solution for six hours with and without Expired Tilmicosin Drug before the experiment started. Certain peaks in Fig. (11) characterize the Expired Tilmicosin Drug’s functional groups. Numerous bands were seen on the spectrum, including the broad band that detected (OH) at 3375 cm^−1^, the band that detected (C = C) at 1644 cm^−1^, and the sharp band that corresponded to (C-O) at 1078 cm^−1”^. A slight change in a few functional groups suggests that the C-steel and Expired Tilmicosin Drug are interacting^64^.

### Quantum chemical parameters

**Fig. 12:**
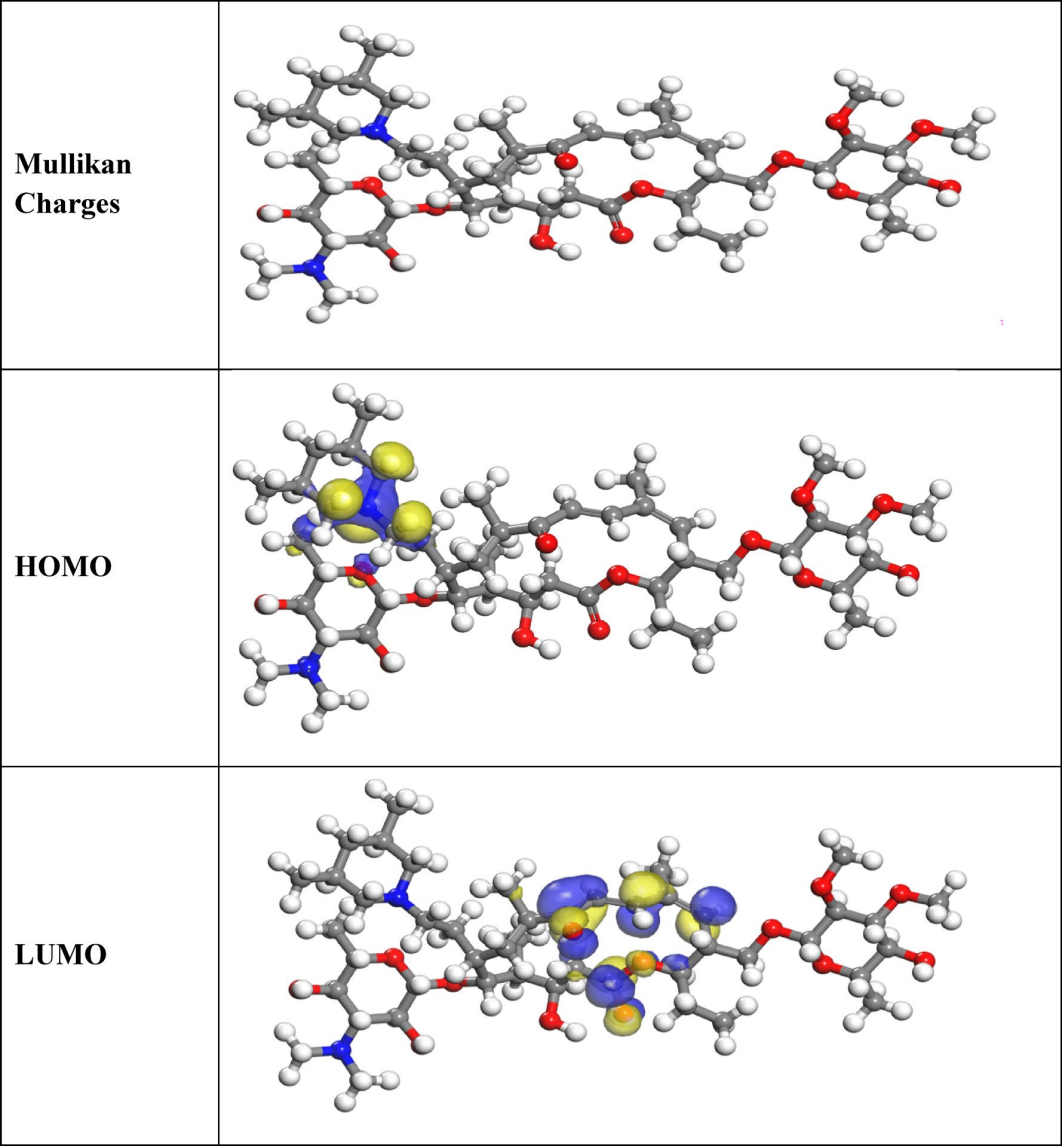
Frontier molecular orbital of expired tilmicosin drug inhibitor (HOMO and LUMO).

According to the DMol3 module built within the Materials Studio version 7.0 software, quantum chemistry was used for all calculations in the current study. “Fig. 12 displays the examination of the inhibitors’ optimal geometry, highest occupied molecular orbital E_HOMO_ denotes the ability of the molecule to donate electron, whereas E_LUMO_ describes the ability of the molecule to accept electron. Thus, the highest value of E_HOMO_ refer to a major affinity for the donation of electrons to unoccupied molecular orbital that was d-orbital of iron atoms and lowest unoccupied molecular orbital (LUMO) density distributions. HOMO and LUMO can determine the donation-acceptance capacity and the molecular reactivity of the Expired Tilmicosin Drug. The inhibition efficiency increases with an increase in EHOMO values along with a decrease in ELUMO values. The increasing values of EHOMO imply a superior tendency to donate electrons to the molecule with empty orbitals. The dipolar moment (µ) is a measure of the polarity with the covalent bond. The energy band gap ΔE_g_ was defined as:ΔE = E_HOMO_ − E_LUMO_. A smaller ΔE value indicates greater molecular reactivity. Generally, a smaller energy gap means a molecule can more easily donate or accept electrons, which facilitates stronger adsorption onto the metal. Therefore, a lower energy gap value corresponds to a highly reactive molecule with good corrosion inhibition efficiency on the metal surface^65^. Table 7 displays the calculated dipole moments (µ), which indicate the polarity of the covalent bonds within the studied compounds. The higher µ value of Expired Tilmicosin Drug (17.27 debye) suggests greater asymmetry in its charge distribution. This larger dipole moment increases Expired Tilmicosin Drug’s polarity, leading to stronger electrostatic interactions between the inhibitor and the charged C-steel surface in acidic HCl media.

**Table 7:** Parameter gotten from quantum for expired tilmicosin drug.

Parameters (Variable)	DFT
E_HOMO,_ (ev)	−4.453
E_LUMO,_ (ev)	−3.309
∆E, (eV), (E_L_-E_H_)	1.144
µ (debye), (Dipole moment)	17.27

### Monte Carlo (MC) simulation

**Fig. 13:**
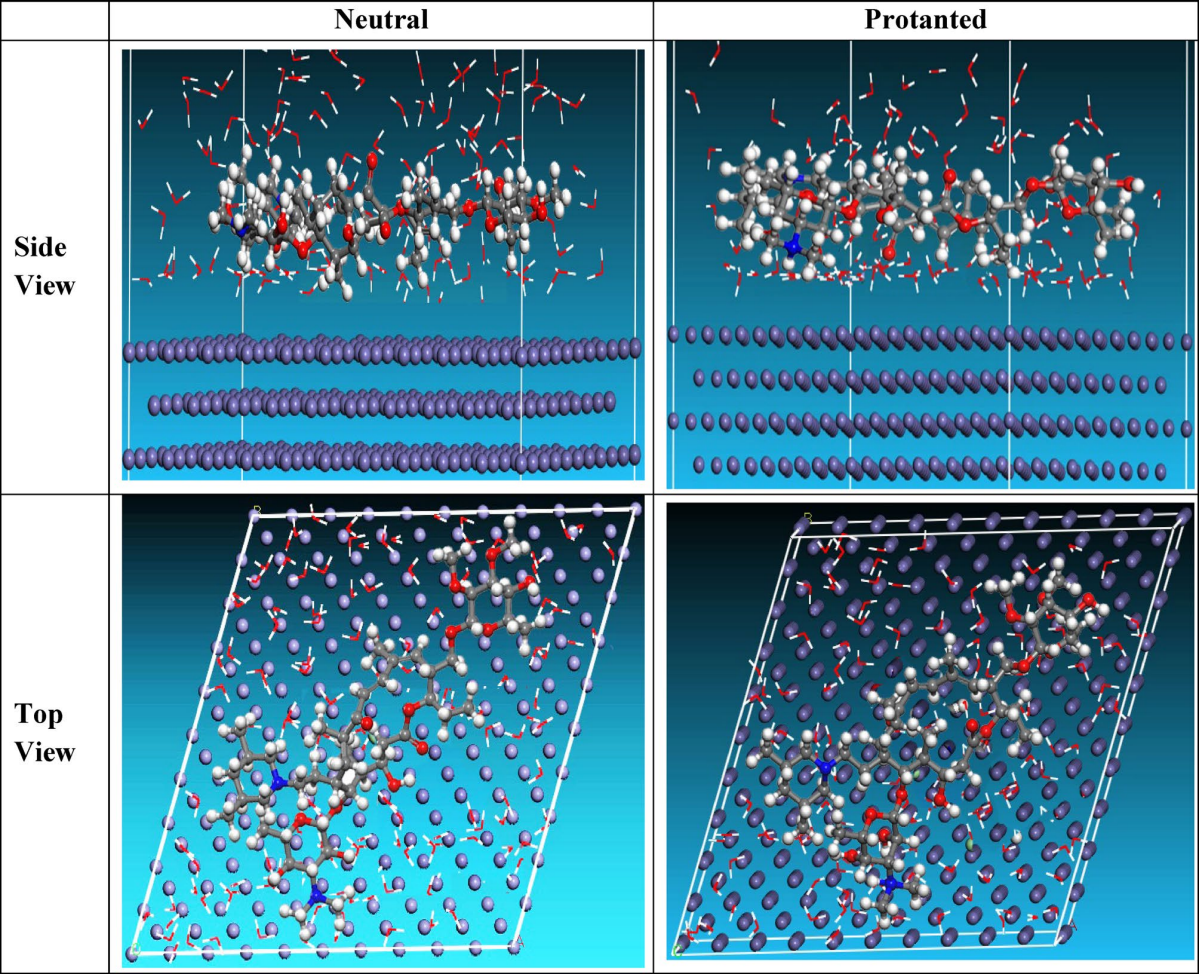
The most appropriate conformation for the adsorption of the expired tilmicosin drug molecule on Fe (1 1 0).

The side and top observations of the most suitable adsorption formations for the “Expired Tilmicosin Drug tested on C-steel surface obtained from the adsorption locator module are thus shown in Fig. 13. Adsorption energy is characterized as declining energy, when materials are mixed during the adsorption process in which an electron, ion, or molecule (adsorbent) is bound to the solid surface. Table 8 shows that Expired Tilmicosin Drug has a greater adsorption energy, indicating that unused Expired Tilmicosin Drug will heavily adsorb on the toughened surface of C-steel to form adsorbed stable layers that will prevent corrosion^66^. ”.

**Table 8:** Results and descriptors measured by the Monte Carlo simulation for adsorption of expired tilmicosin drug molecule on iron (1 1 0).

Structures	Adsorption energy	Rigid adsorption energy	Deformation energy	Compound dE_ad_/dNi	H_2_O dE_ad_/dNi
Fe (1 1 0)/Expired Tilmicosin Drug,/H_2_O	−1514.401	−1578.971	64.57	−87.87	−11.51
Fe (1 1 0)/Protanted Expired Tilmicosin Drug,/H_2_O	−1640.271	−1701.741	61.47	−55.48	−10.88

This specific, tilted orientation increases surface coverage because the phenyl methoxy groups enhance molecular adsorption to the copper surface. This configuration, leading to greater surface coverage than other orientations, improves the overall inhibition efficiency (η). These findings support previous research in this study, which indicates that improved surface coverage results in more effective corrosion protection. Table 7 quantitatively illustrates this interaction. The most favorable adsorption interactions are associated with the lowest adsorption energies, which correspond to the most stable configurations observed in the system. The adsorption energy of Expired Tilmicosin Drug is calculated as −1514.401 kJ mol-1,comparedto − 1640.271 kJ mol-1 for its protonated form. Both protonated and unprotonated Expired Tilmicosin Drug forms interact strongly with the surface. However, the protonated form exhibits stronger inhibitory efficiency due to its more negative (higher magnitude) adsorption energy. The greater magnitude of adsorption energies in the aqueous phase indicates stronger adsorption interactions compared to the gas phase, as solvation effects in water enhance the stabilization of the inhibitor-metal complex. The results demonstrated that Expired Tilmicosin Drug with its higher dipole moment, lower HOMO-LUMO gap, and greater electron-donating ability, exhibits superior adsorption and interaction with the Cu(111) surface. This is further supported by the adsorption energies obtained from Monte Carlo simulations,

#### Inhibition mechanism

In 1 M aqueous HCl, the C-steel surface tends to become positively charged due to the presence of excess hydrogen ions. Several factors determine the adsorption mechanism of Expired Tilmicosin Drug molecules on C-steel surfaces. These include surface conditions, the molecular structure of the inhibitor, and the presence of heteroatoms, as noted in reference^67^. When Expired Tilmicosin Drug is absent, the C-steel’s protective layer is destroyed, leading to the production of Fe^2+^ ions. Adsorption isotherm studies show that Expired Tilmicosin Drug’s adsorption is a mixed-mode process, encompassing both physical (electrostatic) and chemical (charge sharing) interactions. Therefore, the existence of cooperative adsorption between the cationic species and Cl ions may account for the good inhibitive capabilities of the expired tilmicosin drug in HCl. Cooperative adsorption occurs when Cl ions are already adsorbed on the metal surface and Expired Tilmicosin Drug cations are adsorbed there. Because the negative ends of the halide metal dipoles are oriented toward solution, an intermediate bridge may form, increasing the adsorption of the drug cations on the dipoles and producing a positive synergistic effect. This could explain the feasible adsorption of Expired Tilmicosin Drug cations in the presence of Cl ions^68^ This is an example of drug cations physisorbing onto a Cl bridge that has formed on the surface of C-steel. However, in competitive adsorption, the cationic species may compete with the Cl ions for adsorption on various steel surface locations due to the high bulk concentration of the drug cations at high inhibitor concentrations. In this instance, chemisorption i.e. donor-acceptor interaction between the de-localized π-electrons of the rings and the unoccupied low energy d-orbitals of Fe surface atoms is responsible for the adsorption of inhibitor species. The electron density of the rings is significantly increased by the presence of hydroxyl groups, N, and O atoms, which have electron donation capabilities.

## Conclusions

i) Expired Tilmicosin Drug considered as green corrosion inhibitor as it is a nontoxic inhibitor to human in addition, prevention of the steel from corrosion is of environmental importance, as corrosion a terrible waste of both natural resources and money.
ii) As the drug dose increases, the inhibitory efficiency values rise, on the other hand, it decreases by raising temperature.
iii) Expired Tilmicosin Drug study indicates that the medication functions as a mixed-type inhibitor but primarily physically adsorbed according to % IE and thermodynamic parameters.
iv) At all temperatures tested, the Langmuir adsorption isotherm is satisfied.
v) The data obtained from different analytical techniques are in good agreement to each other to indicate that the addition of Expired Tilmicosin Drug inhibits the corrosion of steel in acidic environment and decrease the iron dissolution process in this environment.
vi) This study highlights the utility of DFT and Monte Carlo simulations in evaluating the corrosion inhibition efficiency of Expired Tilmicosin Drug on C-steel surfaces.

## Data Availability

No datasets were generated or analysed during the current study.
